# PERSPECTIVES OF CAREGIVERS OF RURAL, OLDER VETERANS ON TELEGERIATRICS CARE

**DOI:** 10.1093/geroni/igac059.023

**Published:** 2022-12-20

**Authors:** Jacqueline Boudreau, Jennifer Conti, Meaghan Kennedy, Camilla Pimentel, William Hung, Lauren Moo, Eileen Dryden

**Affiliations:** VA Bedford Health Care System, Bedford, Massachusetts, United States; VA Bedford Health Care System, Bedford, Massachusetts, United States; VA Bedford Health Care System, Bedford, Massachusetts, United States; VA Bedford Healthcare System, Bedford, Massachusetts, United States; Icahn School of Medicine at Mount Sinai, New York, New York, United States; VA Bedford Health Care System, Bedford, Massachusetts, United States; VA Bedford Health Care System, Bedford, Massachusetts, United States

## Abstract

Telemedicine is critical to extending healthcare’s reach to rural older adults with complex medical needs, yet concerns remain about feasibility and acceptability for this population and their caregivers. We interviewed 30 rural Veterans ≥65 years old and/or their caregivers (n=21) about their experiences with video or telephone visits as part of an evaluation of Virtual Geriatrics, a network of Veterans Affairs tele-geriatric care hubs. Interviews were recorded, transcribed, and analyzed using rapid qualitative analysis. Caregivers deemed telemedicine a convenient option that prevented burdensome travel to remote specialists, facilitated caregiver involvement in visits, and matched quality of in-person visits. Caregivers often managed technology, enabling their loved one to participate in video visits. Telephone visits, while convenient, sometimes caused missed physical cues and hearing challenges which led providers to lean on caregiver communication. Our findings suggest telemedicine is feasible and acceptable for delivery for geriatrics care among rural adults and their caregivers.

